# The development of a therapeutic strategy for post-acute sequelae of COVID-19 should be based on an efficient classification of pathogenesis

**DOI:** 10.3389/fmed.2025.1718460

**Published:** 2026-01-06

**Authors:** Heng Wang, Li Shen, Song Xue

**Affiliations:** Department of Cardiovascular Surgery, Renji Hospital, Shanghai Jiao Tong University School of Medicine, Shanghai, China

**Keywords:** coinfection, COVID-19, etiology, hypersensitivity, inflammation, persistent infection, SARS-CoV-2

## Abstract

The treatment of post-acute sequelae to COVID-19 (PASC) remains challenging. Defining PASC solely based on symptoms and disease duration in clinical trials can mask the potential for recovery in specific patient subgroups. A good design for future research requires a clear classification of various PASC according to different pathogenesis under the general diagnosis. Here, we discuss four key types of pathogenesis that should be recognized to determine the enrollment of PASC patients.

## 1 Introduction

Patients with coronavirus disease 2019 (COVID-19) can have persistent health issues, including new-onset or enhanced symptoms or signs that are beyond the course of the acute disease after severe acute respiratory syndrome coronavirus-2 (SARS-CoV-2) infection. These conditions are referred to as post-acute sequelae/syndrome of COVID-19 (PASC), long-term condition/complication of COVID-19, long COVID, etc.

The average symptom duration of the COVID-19 acute phase in mild to moderate cases, which is characterized by fever and respiratory symptoms, is 10 days, and the viral RNA shedding can no longer be detected using a nasopharyngeal swab in 2–4 weeks in most cases (1). The incidence of PASC is heterogeneous and estimated to be 10%–15% (2). A widely accepted definition of long COVID can be interpreted as “a condition lasts for at least 2 months, usually 3 months after the acute symptom onset, which is presumably caused by SARS-CoV-2 infection and cannot be explained by an alternative diagnosis” (3). The definition from the U.S. Centers for Disease Control and Prevention regards persistent conditions over 4 weeks after symptom onset with uncharacteristic viral test and antibody profile as late sequelae of COVID-19 (4). Approximately 70%–90% of certain PASC conditions can be recovered in 1 year, but are able to cause a delayed resumption of study (5, 6). There are patients suffering from PASC for a longer period. A considerable prevalence of PASC could still be found 3 years after the symptom onset (7).

Currently, PASC represents a group of symptom-centered conditions with mixed subjects investigated, which baffles standard evaluation and therapy. A classification of PASC based on the underlying disease pathogenesis can promote precision medicine. Here, we discuss four types of PASC pathogenesis based on the disease course, with typical examples that require different therapeutic strategies.

## 2 PASC pathogenesis

### 2.1 Persistent SARS-CoV-2 infection

Persistent SARS-CoV-2 infection, resulting from immune compromise and viral reservoirs in tissues, is not uncommon and is a suspected cause of PASC (8).

It was estimated that 0.7%–3.5% and 0.5%–1% infections can last persistently for at least 30 days and 60 days, respectively, and a risk of more than 50% higher odds of long COVID-19 was found in these cases compared with non-persistent ones (9). The commonly used approaches for pathogen detection, e.g., rapid antigen test for virus antigens and reverse transcription polymerase chain reaction for virus RNA, usually collect samples from the upper respiratory tract, which underestimates the incidence or prevalence of persistent infections in the population. In autopsies, SARS-CoV-2 RNA was found in the vagus nerve, providing evidence of virus-induced inflammation that can cause symptoms related to autonomic nervous system disorders, e.g., postural orthostatic tachycardia syndrome after COVID-19 (10, 11). A study reported an elevation of SARS-CoV-2 antigens by 10.6, 8.7, and 5.4% between 3 and 6 months, between 6 and 10 months, and between 10 and 14 months after diagnosis, respectively, compared with pre-pandemic samples in plasma, and the replicating virus was hypothesized to hide in organs and seed viral components through the bloodstream (12). COVID-19-related myocarditis can happen weeks or months after the acute phase (13). Although evidence suggested that myocarditis in the post-acute period is largely caused by inflammation, virus infection in cardiomyocytes, endothelial cells, and macrophages can last for up to 18 months, which is consistent with the conception of persistent infection (14).

Given that the virus is still active, this type of PASC can be considered a continuous remnant infection after the acute phase.

### 2.2 Secondary infection

Secondary infection is a commonly reported complication that constitutes PASC.

Immune deficiency is a featured perturbation found in long COVID-19 patients, and manifestations caused by evidenced secondary infection should be defined as a unique PASC (15). In a cohort focusing on allergic diseases after the acute COVID-19 phase, an increased hazard of Aspergillus pneumonia was found (16). The increased prevalence of fungal infections during the pandemic was suspected to be a result of both SARS-CoV-2-induced immune deficiency and iatrogenic factors, e.g., using immunomodulatory agents and invasive procedures (17). In another large cohort with 2.4 million cases, researchers found that COVID-19 patients had a higher risk of 1.59 hazard ratio for developing herpes zoster at a 1-year follow-up (18). The activation of Epstein–Barr virus was suspected to be the cause of myalgic encephalomyelitis or chronic fatigue syndrome (ME/CFS) in PASC (19).

Secondary infection initiates a new disease course parallel to COVID-19 and its sequelae, which can be diagnosed as PASC for a period overlapping.

### 2.3 Prolonged recovery

The homeostasis of the internal environment is believed to be stabilized by the neurologic system, immune system, and endocrine system, forming a neuro-immuno-endocrinologic regulating net (20). The internal environment alteration can continue both when virus replication is active and after virus clearance. There are several mechanisms leading to such a prolonged recovery.

#### 2.3.1 Hypersensitivity

Hypersensitivity, a mechanism underlying autoimmune diseases, also plays a significant role in PASC (21). There are four types of hypersensitivity, (1) type 1, mediated mainly by mast cells and immunoglobulin E (IgE), which is usually addressed as anaphylaxis; (2) type 2, mediated mainly by IgM and IgG, leading to tissue damage (2a) or stimulating reactions (2b); (3) type 3, mediated mainly by immune complexes, leading to local inflammation and tissue damage; and (4) type 4, mediated by T cells, and is divided into four sub-types according to the T cell subgroups (22). Hypersensitivity can be induced when SARS-CoV-2 replication and invasion are active and may continue to exert its effect for a long period after the virus turns negative. The duration of hypersensitivity-induced conditions can fall into the definitive range of PASC. Studies have reported a significant increase in the 30-day hazard of asthma, a typical type 1 hypersensitivity disease, along with other allergic diseases among COVID-19 patients compared with the general population and influenza patients (16, 23). Multi-system inflammatory syndrome that usually occurs in children (MIS-C) weeks or months after the acute phase can be mediated by immunopathology of cytokine storm and autoimmune response, where evidence has illustrated auto-antibodies interacting with endothelium, immune cells, and other host tissues (type 2a hypersensitivity), and immune complexes (type 3 hypersensitivity) trigger inflammation (24). In addition, cytotoxic T cell-induced β cell apoptosis is a typical type 4 hypersensitivity resulting in type 1 diabetes, which may contribute to the increased incidence of type 1 diabetes during the pandemic, as the relationship between type 1 diabetes and SARS-CoV-2 infection appears to be speculative (25, 26).

#### 2.3.2 Cytokine-induced endocrine disruption

Some endocrine disruption can result from factors other than hypersensitivity. The inflammatory status can directly affect the endocrine organs. Hypothalamic–pituitary–adrenal axis plays a central role in multiple long COVID conditions, including cardiopulmonary dysfunction, musculoskeletal diseases, and gastrointestinal symptoms (27). SARS-CoV-2 can lead to arginine vasopressin release stimulated through elevated pro-inflammatory cytokines and dysregulates the hypothalamic–pituitary–adrenal axis (28, 29). Although SARS-CoV-2 vaccination can ameliorate thyroid dysfunction after infection, which indicates a causal influence of virus-induced abnormality, autoimmunity may not be the dominating mechanism with few evidence, and cytokine storm remains to be a validated explanation (30–32). IL1β, IL6, TNF-*α*, and IFN-*γ* secreted by dendritic cells and mononuclear macrophages are key mediators of thyroid dysfunction (33). Cytokines can also directly inhibit the secretion of melatonin from the pineal gland, causing disruption of the circadian rhythm (34). The duration for these endocrine disruptions to resolve after the primary inflammatory response to viral infection is unclear.

#### 2.3.3 Hyper-coagulation

The coagulation and fibrinolysis system keeps a fragile balance in inflammation (35). The hyper-coagulation with Virchow’s triad, i.e., endothelial injury, abnormal blood flow, and hypercoagulability, can lead to an increased risk of thromboembolic diseases, e.g., pulmonary embolism and stroke (36). Currently, (1) activation of coagulating factors by inflammation and (2) virus direct cytopathic effect have been well concluded (37, 38), while the blood flow change may be underestimated in PASC hypercoagulation. A meta-analysis of large cohorts revealed that SARS-CoV-2 can twice the risk of atrial fibrillation, the main cause of stroke, in the recovered (39). In severe cases with long-term immobility and individuals suffering from ME/CFS, deep vein thrombosis can also be a life-threatening complication (40).

#### 2.3.4 Microbiome imbalance

Growing evidence indicates that the gut microbiome can regulate the internal environment. The gut–brain axis alteration can cause ME/CFS, a common disease in PASC, through immune, neurological, and metabolic axes, and targeted interventions have shown considerable therapeutic efficacy (41). The microbiome can also be changed by SARS-CoV-2 infection or therapy using glucocorticoids for inflammation control and antibiotics for antibacterial treatment (42–45). The characterized microbiome change in increased *Bacteroides*, *Flavonifractor*, *Ruminococcus gnavus*, and decreased *Bifidobacterium*, *Dorea*, and *Faecalibacterium prausnitzii*, has been observed consistently in PASC patients (42). The emerging field of lung microbiome may provide novel choices for future research in PASC development (46). However, more discovery is needed to develop corresponding therapies for PASC with digital health systems, data science, and bioinformatics.

### 2.4 Incomplete recovery

Some PASC cases cannot be explained by active infection or undergoing pathophysiology, and their symptoms may result from permanent tissue damage, namely, incomplete recovery.

In the acute phase, SARS-CoV-2 causes severe inflammation that activates several fibrogenic cell pathways and biological axes, leading to fiber deposition (47). This pathological change can irreversibly impair respiratory function. Along with diagnosed diffusion capacity impairment and restrictive pulmonary dysfunction, persistent ground-glass opacities and pulmonary fibrosis in COVID-19 patients did not show recovery in a 1-year follow-up (48). An increased risk of acute myocardial infarction can be found in COVID-19 patients at the 8.5-month follow-up, which can also cause permanent cardiac dysfunction, as cardiomyocytes are not renewable (49).

In these cases, the course of COVID-19 reaches an end where no active infection or internal environment alteration can be reversed with currently available clinical interventions.

## 3 Challenges

### 3.1 Mechanism overlapping

Research in basic medicine and biology typically focuses on disease etiology; however, this approach may struggle to distinguish between mechanistic “sets” or “levels” due to their frequent intersection. Efforts were made to classify PASC pathogenesis on some bases. A common framework for describing disease mechanisms is structured around molecular and biological processes and their corresponding morphological (pathological) and functional (pathophysiological) manifestations (50, 51). Anatomical systems based on physiological functions are also a widely used view, which analyzes the pathological changes of each system with little overlapping and provides explanations for the chief complaints (52, 53). With the use of modern biomedical technologies, molecular patterns with machine learning algorithms have become a reliable view for classification, helping to elucidate the biological basis of PASC (54).

**Figure 1:**
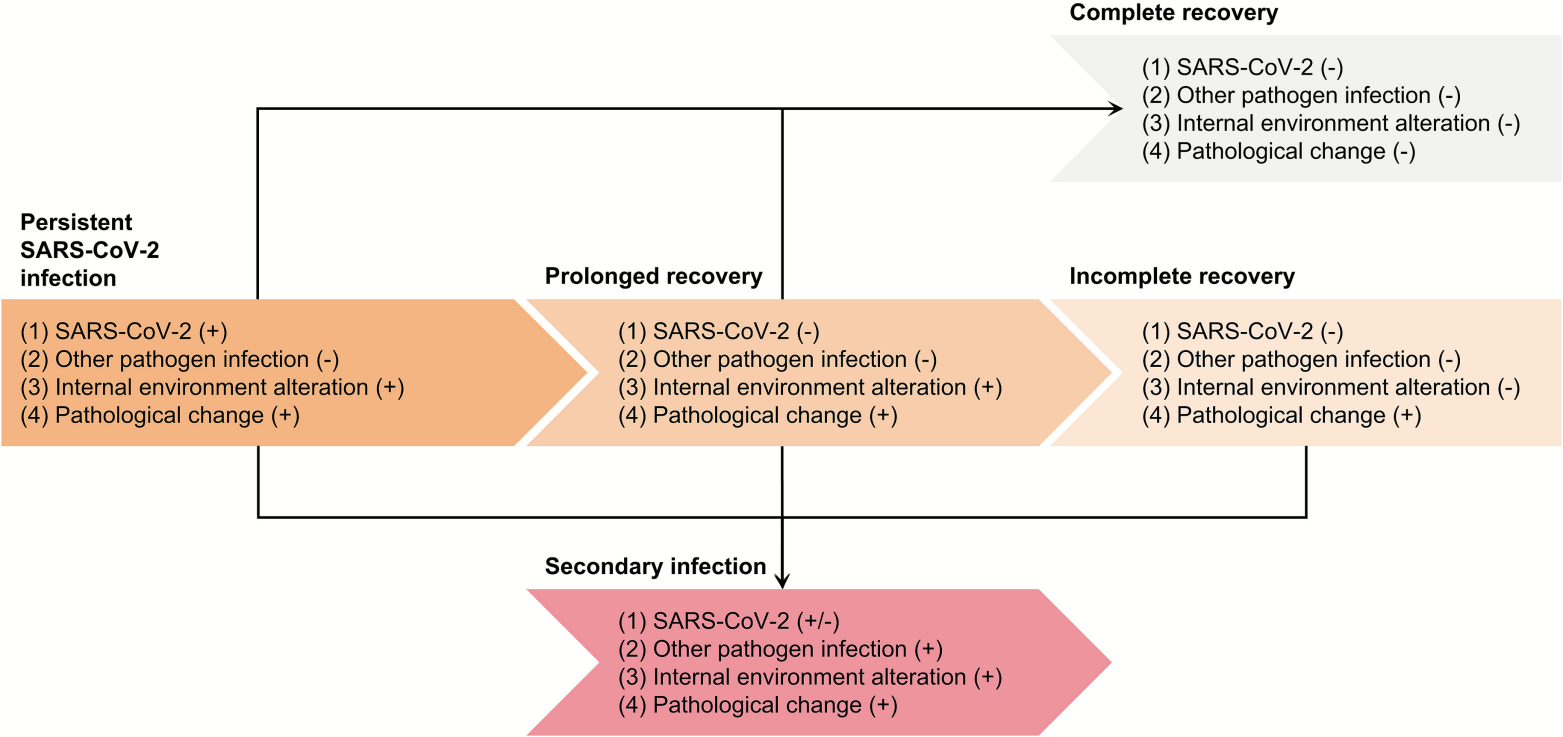
Classification of PASC based on pathogenesis. The subtypes of PASC are determined by three characters: SARS-CoV-2 positivity, secondary infection, and reversible internal environment alteration. SARS-CoV-2, severe acute respiratory syndrome coronavirus-2.

Basically, in the disease course, there are two clinical outcomes, recovery and death, where recovery is divided into incomplete recovery and complete recovery. Here, we define the word “prolonged recovery” as a continuous, active disease status that does not reach a clinical outcome (recovery or death), and “incomplete recovery” as one of the clinical outcomes. Our disease course-based classification of PASC groups patients into the following categories: non-recovered (prolonged recovery, with specified subcategories for viral persistence and secondary infection), and incompletely recovered. This system provides a basis for clinical trials by enabling the initial stratification of patients by the underlying pathogenesis, thus minimizing confounding (Figure 1).

### 3.2 Diagnostic uncertainty

**Figure 2:**
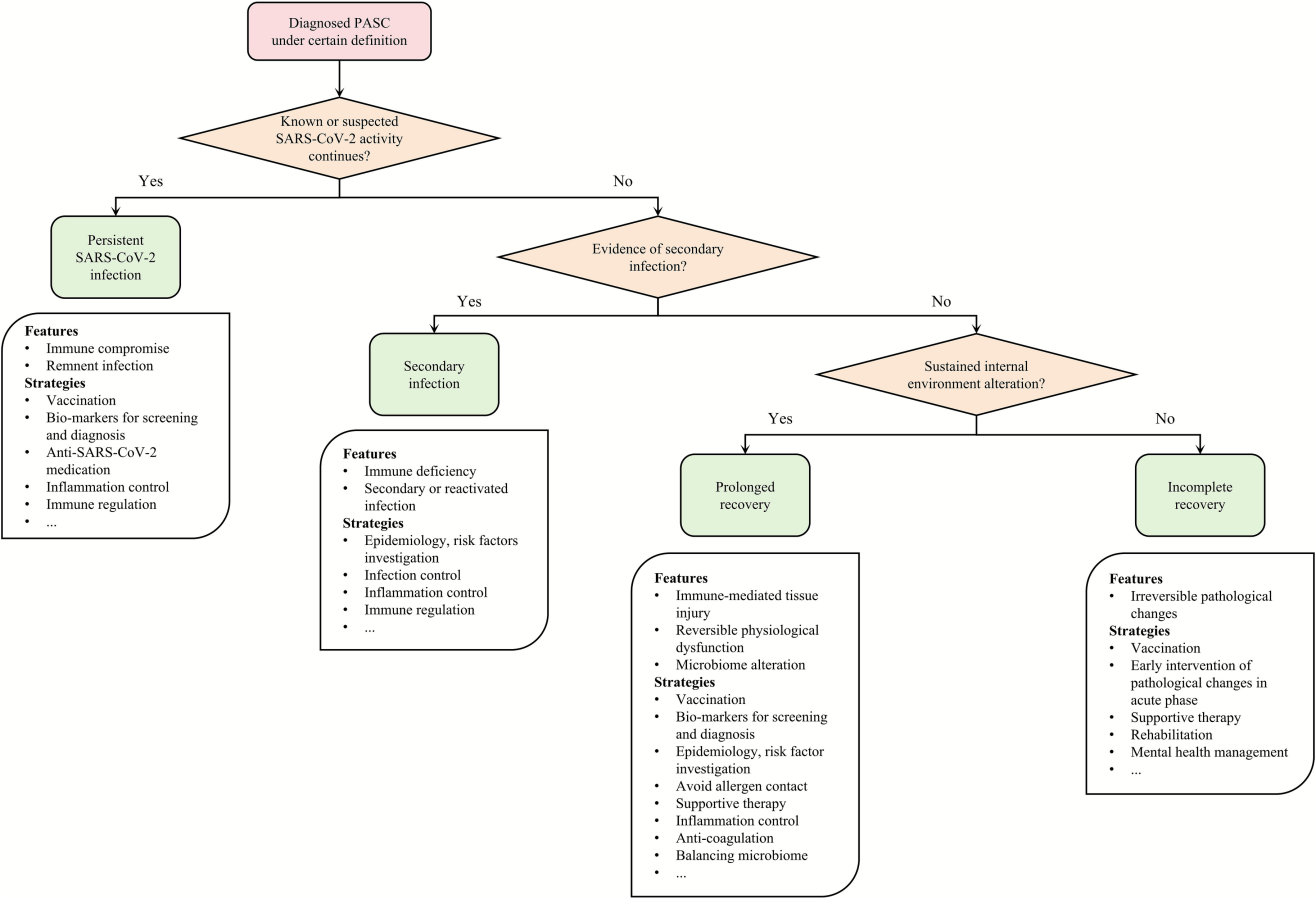
Referenced workflow for patient enrollment. The flowchart illustrates the analysis and determination of PASC for patient enrollment with corresponding strategies for future investigations into prevention, diagnosis, therapy, and long-term management. PASC, post-acute syndrome of coronavirus disease 2019; SARS-CoV-2, severe acute respiratory syndrome coronavirus-2.

More studies are needed for the individual identification of pathogenesis. After the initial diagnosis of PASC, whether SARS-CoV-2 is still active should be confirmed. Since the upper respiratory tract may test negative in persistent infection, further testing for biopsies, plasma, excreta, etc., may be considered (55). If direct evidence of virus existence cannot be obtained, risk assessment based on autopsy and epidemiological factors may be carried out. Given that co-infections occur in approximately 26% of COVID-19 patients (56), secondary infections likely constitute a substantial proportion of PASC mechanisms; however, the spectrum of secondary infection in PASC patients is yet to be explored. Considering that (1) active SARS-CoV-2 replication and invasion can maintain the immunodeficiency status, and (2) secondary infection is often hard to identify, identification and clearance of SARS-CoV-2 should be prioritized. In prolonged recovery, the mechanisms form a highly interconnected spiderweb, maybe requiring biomarkers with high sensitivity and specificity, including cytokines, immune cells, nutrients, metabolites, and hormones (57, 58), and gut and lung microbiome. Objective results from electro- and/or echocardiography, respiratory examination, ultrasonic, and radiographic imaging should be considered. Only when intervenable changes are excluded can physicians classify the PASC patient into incomplete recovery, to avoid masking underlying recovery (Figure 2).

### 3.3 Clinical implementation

Trials on PASC can enrich severe cases with significant symptoms or signs, as many cases were missed in diagnosis (59). The PASC incidence and severity can also be different among SARS-CoV-2 lineages, e.g., MIS-C severity was highest during the Alpha (B.1.1.7) wave, followed by the Delta (B.1.617.2) wave, and was lowest in the Omicron (B.1.1.529) period (60, 61); the incidence of PASC was estimated to be 10.4, 9.5, 7.7% in Pre-Delta, Delta, and Omicron periods (62). Therefore, epidemiological research on large observational cohorts for the general population is still necessary. It is sometimes unlikely to divide all included PASC patients into the aforementioned pathogenesis types without appropriate diagnosis kits, while specific subgroups with detected remnant SARS-CoV-2 infection, secondary infection, or clear biomarkers indicating internal environment alteration may benefit from precision therapy. Given that PASC is characterized by non-severe baseline disease but remarkable treatment resistance, strict enrollment criteria inevitably shrink the sample size. This elevates the risk of type II statistical error in the outcome analysis. To mitigate this risk, the sample size calculation is indispensable (63). Furthermore, as mental health is an integral yet non-objective component of PASC, it should be formally evaluated with appropriate scales to mitigate the risk of unanticipated confounding (64–66). Considering that some factors in diagnostic results hardly contribute to the probability of necessity, meaning their reversal or re-normalization may not result in a significant reduction in disease severity, causal inference may be carried out before trial enrollment (67, 68).

## 4 Discussion

Targeting persistent SARS-CoV-2 infection is a promising strategy. Although small-molecule antivirals did not show a significant improvement in enrolled PASC patients (69), taking antivirals during the acute phase may reduce the risk of PASC (70), which may be explained by the effective limitation of virus replication and spreading during the acute phase. Consistently, both pre- and post-COVID-19 vaccination reduced the risk of developing PASC (71). The timing and methods of SARS-CoV-2 clearance should be further investigated in PASC.

Some studies have reported promising results when enrollment was based on specific internal environment alteration. In PASC patients with vitamin K2/D3 deficiency, vitamin intake improved the long COVID-19 index (72). The immunomodulatory therapy methylprednisolone and tocilizumab shortened the hospital stay of MIS-C (73). Convalescent patients with endothelial dysfunction benefited from sulodexide, an anti-thrombotic and pro-fibrinolytic drug (74).

Efforts have been made to treat lung fibrosis, and lung function can be improved by anti-fibrotic medications (75). However, most incomplete recoveries may need physical exercise and other rehabilitation (76).

In conclusion, PASC diagnosis based on symptoms and the duration of disease is commonly used to determine the inclusion criteria of clinical trials. However, PASC varies among different populations, making enrolled cases heterogeneous, thus masking the potential for recovery in subgroups of specific pathogenesis. Developing strategies according to PASC pathogenesis provides causal inference insights for effective ways of controlling symptoms and signs.

## Data Availability

The original contributions presented in the study are included in the article/supplementary material, further inquiries can be directed to the corresponding authors.

## References

[1] Puhach O Meyer B Eckerle I. Sars-Cov-2 viral load and shedding kinetics. Nat Rev Microbiol. (2023) 21:147–61. doi: 10.1038/S41579-022-00822-W, 36460930 PMC9716513

[2] Nalbandian A Desai AD Wan EY. Post-COVID-19 condition. Annu Rev Med. (2023) 74:55–64. doi: 10.1146/Annurev-Med-043021-030635, 35914765

[3] Soriano JB Murthy S Marshall JC Relan P Diaz JV. A clinical case definition of post-COVID-19 condition by a Delphi consensus. Lancet Infect Dis. (2022) 22:E102–7. doi: 10.1016/S1473-3099(21)00703-9 34951953 PMC8691845

[4] Datta SD Talwar A Lee JT. A proposed framework and timeline of the spectrum of disease due to SARS-Cov-2 infection: illness beyond acute infection and public health implications. JAMA. (2020) 324:2251–2. doi: 10.1001/Jama.2020.22717 33206133

[5] Chilunga FP Appelman B Van Vugt M Kalverda K Smeele P Van Es J . Differences in incidence, nature of symptoms, and duration of long COVID among hospitalised migrant and non-migrant patients in the Netherlands: a retrospective cohort study. Lancet Reg Health Eur. (2023) 29:100630. doi: 10.1016/J.Lanepe.2023.100630 37261215 PMC10079482

[6] Yang T Yan MZ Li X Lau E. Sequelae of COVID-19 among previously hospitalized patients up to 1 year after discharge: a systematic review and meta-analysis. Infection. (2022) 50:1067–109. doi: 10.1007/S15010-022-01862-3 35750943 PMC9244338

[7] Rahmati M Udeh R Kang J Dolja-Gore X Mcevoy M Kazemi A . Long-term sequelae of COVID-19: a systematic review and meta-analysis of symptoms 3 years post-Sars-Cov-2 infection. J Med Virol. (2025) 97:E70429. doi: 10.1002/Jmv.70429, 40476637 PMC12143191

[8] Machkovech HM Hahn AM Garonzik Wang J Grubaugh ND Halfmann PJ Johnson MC . Persistent Sars-Cov-2 infection: significance and implications. Lancet Infect Dis. (2024) 24:E453–62. doi: 10.1016/S1473-3099(23)00815-0 38340735

[9] Ghafari M Hall M Golubchik T Ayoubkhani D House T Macintyre-Cockett G . Prevalence of persistent SARS-Cov-2 in a large community surveillance study. Nature. (2024) 626:1094–101. doi: 10.1038/S41586-024-07029-4, 38383783 PMC10901734

[10] Woo MS Shafiq M Fitzek A Dottermusch M Altmeppen H Mohammadi B . Vagus nerve inflammation contributes to dysautonomia in COVID-19. Acta Neuropathol. (2023) 146:387–94. doi: 10.1007/S00401-023-02612-X 37452829 PMC10412500

[11] Ormiston CK Świątkiewicz I Taub PR. Postural orthostatic tachycardia syndrome as a sequela of COVID-19. Heart Rhythm. (2022) 19:1880–9. doi: 10.1016/J.Hrthm.2022.07.014, 35853576 PMC9287587

[12] Peluso MJ Swank ZN Goldberg SA Lu S Dalhuisen T Borberg E . Plasma-based antigen persistence in the post-acute phase of COVID-19. Lancet Infect Dis. (2024) 24:E345–345e347. doi: 10.1016/S1473-3099(24)00211-1 38604216 PMC11650779

[13] Nappi F Avtaar Singh SS. Sars-Cov-2-induced myocarditis: a state-of-the-art review. Viruses. (2023) 15:916. doi: 10.3390/V15040916 37112896 PMC10145666

[14] Blagova O Lutokhina Y Kogan E Kukleva A Ainetdinova D Novosadov V . Chronic biopsy proven post-COVID Myoendocarditis with Sars-Cov-2 persistence and high level of antiheart antibodies. Clin Cardiol. (2022) 45:952–9. doi: 10.1002/Clc.23886, 35855554 PMC9349976

[15] Yin K Peluso MJ Luo X Thomas R Shin MG Neidleman J . Long COVID manifests with T cell dysregulation, inflammation and an uncoordinated adaptive immune response to SARS-Cov-2. Nat Immunol. (2024) 25:218–25. doi: 10.1038/S41590-023-01724-6 38212464 PMC10834368

[16] Choi Y Kim HJ Park J Lee M Kim S Koyanagi A . Acute and post-acute respiratory complications of SARS-Cov-2 infection: population-based cohort study in South Korea and Japan. Nat Commun. (2024) 15:4499. doi: 10.1038/S41467-024-48825-W 38802352 PMC11130304

[17] Singh A Kaur A Chowdhary A. Fungal pathogens and COVID-19. Curr Opin Microbiol. (2023) 75:102365. doi: 10.1016/J.Mib.2023.102365, 37625261

[18] Chen YC Ho CH Liu TH Wu JY Huang PY Tsai YW . Long-term risk of herpes zoster following COVID-19: a retrospective cohort study of 2,442,686 patients. J Med Virol. (2023) 95:E28745. doi: 10.1002/Jmv.28745 37185849

[19] Ruiz-Pablos M Paiva B Zabaleta A. Epstein-Barr virus-acquired immunodeficiency in Myalgic encephalomyelitis-is it present in long COVID. J Transl Med. (2023) 21:633. doi: 10.1186/S12967-023-04515-7, 37718435 PMC10506247

[20] Ponce-Regalado MD Pérez-Sánchez G Rojas-Espinosa O Arce-Paredes P Girón-Peréz MI Pavón-Romero L . Neuroimmunoendocrinology: a brief historic narrative. J Leukoc Biol. (2022) 112:97–114. doi: 10.1002/Jlb.5mr1221-287r 35098580

[21] Yazdanpanah N Rezaei N. Autoimmune complications of COVID-19. J Med Virol. (2022) 94:54–62. doi: 10.1002/Jmv.27292, 34427929 PMC8661629

[22] Dispenza MC. Classification of hypersensitivity reactions. Allergy Asthma Proc. (2019) 40:470–3. doi: 10.2500/Aap.2019.40.4274, 31690397

[23] Oh J Lee M Kim M Kim HJ Lee SW Rhee SY . Incident allergic diseases in post-COVID-19 condition: multinational cohort studies from South Korea, Japan and the UK. Nat Commun. (2024) 15:2830. doi: 10.1038/S41467-024-47176-W 38565542 PMC10987608

[24] Constantin T Pék T Horváth Z Garan D Szabó AJ. Multisystem inflammatory syndrome in children (MIS-C): implications for long COVID. Inflammopharmacology. (2023) 31:2221–36. doi: 10.1007/S10787-023-01272-3 37460909 PMC10518292

[25] Anindya R Rutter GA Meur G. New-onset type 1 diabetes and severe acute respiratory syndrome coronavirus 2 infection. Immunol Cell Biol. (2023) 101:191–203. doi: 10.1111/Imcb.12615, 36529987 PMC9877852

[26] Rahmati M Yon DK LEE SW Udeh R Mcevoy M Kim MS . New-onset type 1 diabetes in children and adolescents as postacute sequelae of Sars-Cov-2 infection: a systematic review and meta-analysis of cohort studies. J Med Virol. (2023) 95:E28833. doi: 10.1002/Jmv.28833, 37264687

[27] Di Filippo L Franzese V Santoro S Doga M Giustina A. Long COVID and pituitary dysfunctions: a bidirectional relationship. Pituitary. (2024) 27:955–69. doi: 10.1007/S11102-024-01442-8, 39240511

[28] Al-Kuraishy HM Al-Gareeb AI Qusti S Alshammari EM Atanu FO Batiha GE. Arginine vasopressin and pathophysiology of COVID-19: an innovative perspective. Biomed Pharmacother. (2021) 143:112193. doi: 10.1016/J.Biopha.2021.112193, 34543987 PMC8440235

[29] Rodriguez L Brodin P. Immune system perturbations in patients with long COVID. Trends Mol Med. (2024) 30:200–1. doi: 10.1016/J.Molmed.2023.12.008, 38177028

[30] Cheng KL Yu WS Wang YH Ibarburu GH Lee HL Wei JC. Long-term thyroid outcomes after COVID-19 vaccination: a cohort study of 2 333 496 patients from the Trinetx network. J Clin Endocrinol Metab. (2025) 110:E3366–75. doi: 10.1210/clinem/dgaf064, 39883558

[31] Muller I Daturi A Varallo M Re TE Dazzi D Maioli S . Long-term outcome of thyroid abnormalities in patients with severe Covid-19. Eur Thyroid J. (2023) 12:E220200. doi: 10.1530/ETJ-22-0200, 36715690 PMC10083670

[32] Lui D Lee CH Woo YC Hung I Lam K. Thyroid dysfunction in COVID-19. Nat Rev Endocrinol. (2024) 20:336–48. doi: 10.1038/s41574-023-00946-w, 38347167

[33] Ruggeri RM Campennì A Deandreis D Siracusa M Tozzoli R Petranović Ovčariček P . SARS-Cov-2-related immune-inflammatory thyroid disorders: facts and perspectives. Expert Rev Clin Immunol. (2021) 17:737–59. doi: 10.1080/1744666x.2021.1932467, 34015983 PMC8182818

[34] Sasikumar S Unniappan S. SARS-Cov-2 infection and the neuroendocrine system. Neuroendocrinology. (2024) 114:1158–75. doi: 10.1159/000542164, 39433026

[35] Levi M Van Der Poll T. Inflammation and coagulation. Crit Care Med. (2010) 38:S26–34. doi: 10.1097/Ccm.0b013e3181c98d21, 20083910

[36] Gonzalez-Gonzalez FJ Ziccardi MR Mccauley MD. Virchow's triad and the role of thrombosis in COVID-related stroke. Front Physiol. (2021) 12:769254. doi: 10.3389/Fphys.2021.769254, 34858214 PMC8631516

[37] Valencia I Lumpuy-Castillo J Magalhaes G Sánchez-Ferrer CF Lorenzo Ó Peiró C. Mechanisms of endothelial activation, hypercoagulation and thrombosis in COVID-19: a link with diabetes mellitus. Cardiovasc Diabetol. (2024) 23:75. doi: 10.1186/S12933-023-02097-8 38378550 PMC10880237

[38] Conway EM Mackman N Warren RQ Wolberg AS Mosnier LO Campbell RA . Understanding Covid-19-associated coagulopathy. Nat Rev Immunol. (2022) 22:639–49. doi: 10.1038/S41577-022-00762-9, 35931818 PMC9362465

[39] Zuin M Ojeda-Fernández L Torrigiani G Bertini M. Risk of incident atrial fibrillation after Covid-19 infection: a systematic review and meta-analysis. Heart Rhythm. (2024) 21:1613–20. doi: 10.1016/J.Hrthm.2024.04.064, 38636931

[40] Schulman S Makatsariya A Khizroeva J Bitsadze V Kapanadze D. The basic principles of pathophysiology of venous thrombosis. Int J Mol Sci. (2024) 25:11447. doi: 10.3390/Ijms252111447, 39519000 PMC11547114

[41] König RS Albrich WC Kahlert CR Bahr LS Löber U Vernazza P . The gut microbiome in myalgic encephalomyelitis (ME)/chronic fatigue syndrome (CFS). Front Immunol. (2021) 12:628741. doi: 10.3389/Fimmu.2021.628741 35046929 PMC8761622

[42] Lau RI Su Q Ng SC. Long COVID and gut microbiome: insights into pathogenesis and therapeutics. Gut Microbes. (2025) 17:2457495. doi: 10.1080/19490976.2025.2457495, 39854158 PMC11776476

[43] King LR. Gastrointestinal manifestations of long COVID. Life Sci. (2024) 357:123100. doi: 10.1016/J.Lfs.2024.123100 39357795

[44] Zang J Jiang L Wang Y Chen Y Fu C Kasprzyk-Hordern B . Impact of easing COVID-19 restrictions on antibiotic usage in eastern China using wastewater-based epidemiology. Nat Commun. (2024) 15:10161. doi: 10.1038/S41467-024-54498-2, 39580546 PMC11585548

[45] Guarnotta V Ferrigno R Martino M Barbot M Isidori AM Scaroni C . Glucocorticoid excess and COVID-19 disease. Rev Endocr Metab Disord. (2021) 22:703–14. doi: 10.1007/S11154-020-09598-X 33025384 PMC7538187

[46] Li R Li J Zhou X. Lung microbiome: new insights into the pathogenesis of respiratory diseases. Signal Transduct Target Ther. (2024) 9:19. doi: 10.1038/S41392-023-01722-Y, 38228603 PMC10791971

[47] Hirawat R Jain N Aslam Saifi M Rachamalla M Godugu C. Lung fibrosis: post-COVID-19 complications and evidences. Int Immunopharmacol. (2023) 116:109418. doi: 10.1016/J.Intimp.2022.109418, 36736220 PMC9633631

[48] Lee JH Yim JJ Park J. Pulmonary function and chest computed tomography abnormalities 6-12 months after recovery from COVID-19: a systematic review and meta-analysis. Respir Res. (2022) 23:233. doi: 10.1186/S12931-022-02163-X, 36068582 PMC9446643

[49] Zuin M Rigatelli G Battisti V Costola G Roncon L Bilato C. Increased risk of acute myocardial infarction after COVID-19 recovery: a systematic review and meta-analysis. Int J Cardiol. (2023) 372:138–43. doi: 10.1016/J.Ijcard.2022.12.032, 36535564 PMC9755219

[50] Mehandru S Merad M. Pathological sequelae of long-haul COVID. Nat Immunol. (2022) 23:194–202. doi: 10.1038/S41590-021-01104-Y, 35105985 PMC9127978

[51] Perumal R Shunmugam L Naidoo K Wilkins D Garzino-Demo A Brechot C . Biological mechanisms underpinning the development of long COVID. Iscience. (2023) 26:106935. doi: 10.1016/J.Isci.2023.106935, 37265584 PMC10193768

[52] Castanares-Zapatero D Chalon P Kohn L Dauvrin M Detollenaere J Maertens De Noordhout C . Pathophysiology and mechanism of long COVID: a comprehensive review. Ann Med. (2022) 54:1473–87. doi: 10.1080/07853890.2022.2076901, 35594336 PMC9132392

[53] Gheorghita R Soldanescu I Lobiuc A Caliman Sturdza OA Filip R Constantinescu-Bercu A . The knowns and unknowns of long COVID-19: from mechanisms to therapeutical approaches. Front Immunol. (2024) 15:1344086. doi: 10.3389/Fimmu.2024.1344086, 38500880 PMC10944866

[54] Constantinescu-Bercu A Lobiuc A Căliman-Sturdza OA Oiţă RC Iavorschi M Pavăl NE . Long COVID: molecular mechanisms and detection techniques. Int J Mol Sci. (2023) 25:408. doi: 10.3390/Ijms25010408 38203577 PMC10778767

[55] Proal AD Aleman S Bomsel M Brodin P Buggert M Cherry S . Targeting the SARS-Cov-2 reservoir in long COVID. Lancet Infect Dis. (2025) 25:e294–306. doi: 10.1016/S1473-3099(24)00769-2 39947217 PMC12151306

[56] Suleiman AS Islam MA Akter MS Amin MR Werkneh AA Bhattacharya P. A meta-meta-analysis of co-infection, secondary infections, and antimicrobial resistance in Covid-19 patients. J Infect Public Health. (2023) 16:1562–90. doi: 10.1016/J.Jiph.2023.07.005, 37572572

[57] Thomas C Faghy MA Chidley C Phillips BE Bewick T Ashton RE. Blood biomarkers of long COVID: a systematic review. Mol Diagn Ther. (2024) 28:537–74. doi: 10.1007/S40291-024-00731-Z, 39103645

[58] Espín E Yang C Shannon CP Assadian S He D Tebbutt SJ. Cellular and molecular biomarkers of long COVID: a scoping review. EBioMedicine. (2023) 91:104552. doi: 10.1016/J.Ebiom.2023.104552, 37037165 PMC10082390

[59] Gu L Yue J Lin J Liu Z Huang JA. Challenges in diagnosis and treatment of long COVID. Front Med (Lausanne). (2025) 12:1641411. doi: 10.3389/Fmed.2025.1641411, 40917843 PMC12408318

[60] Levy N Koppel JH Kaplan O Yechiam H Shahar-Nissan K Cohen NK . Severity and incidence of multisystem inflammatory syndrome in children during 3 SARS-Cov-2 pandemic waves in Israel. JAMA. (2022) 327:2452–4. doi: 10.1001/Jama.2022.8025, 35588048 PMC9121298

[61] Mccrindle BW Harahsheh AS Handoko R Raghuveer G Portman MA Khoury M . SARS-Cov-2 variants and multisystem inflammatory syndrome in children. N Engl J Med. (2023) 388:1624–6. doi: 10.1056/Nejmc2215074, 36947454 PMC10052214

[62] Xie Y Choi T Al-Aly Z. Postacute sequelae of SARS-Cov-2 infection in the pre-Delta, Delta, and omicron eras. N Engl J Med. (2024) 391:515–25. doi: 10.1056/Nejmoa2403211, 39018527 PMC11687648

[63] Wang X Ji X. Sample size estimation in clinical research: from randomized controlled trials to observational studies. Chest. (2020) 158:S12–20. doi: 10.1016/J.Chest.2020.03.010, 32658647

[64] De Figueiredo CS Sandre PC Portugal L Mázala-De-Oliveira T Da Silva Chagas L Raony Í . COVID-19 pandemic impact on children and adolescents' mental health: biological, environmental, and social factors. Prog Neuro-Psychopharmacol Biol Psychiatry. (2021) 106:110171. doi: 10.1016/J.Pnpbp.2020.110171, 33186638 PMC7657035

[65] Sankar K Gould MK Prescott HC. Psychological morbidity after COVID-19 critical illness. Chest. (2023) 163:139–47. doi: 10.1016/J.Chest.2022.09.035, 36202259 PMC9528063

[66] Munipalli B Al-Soleiti M Morris A Rummans T. COVID-19: ramifications of the pandemic on mental health and substance abuse. Front Public Health. (2024) 12:1401734. doi: 10.3389/Fpubh.2024.1401734, 39145172 PMC11322968

[67] Alcalá-Santiago Á Rodriguez-Barranco M Sánchez MJ Gil Á García-Villanova B Molina-Montes E. Micronutrients, vitamin D, and inflammatory biomarkers in COVID-19: a systematic review and meta-analysis of causal inference studies. Nutr Rev. (2025) 83:E1383–1383e1405. doi: 10.1093/Nutrit/Nuae152, 39449666 PMC12166185

[68] Zhou D Gamazon ER. Integrative transcriptomic, evolutionary, and causal inference framework for region-level analysis: application to COVID-19. NPJ Genom Med. (2022) 7:24. doi: 10.1038/S41525-022-00296-Y, 35318325 PMC8940898

[69] Geng LN Bonilla H Hedlin H Jacobson KB Tian L Jagannathan P . Nirmatrelvir-ritonavir and symptoms in adults with postacute sequelae of SARS-CoV-2 infection: the STOP-PASC randomized clinical trial. JAMA Intern Med. (2024) 184:1024–34. doi: 10.1001/Jamainternmed.2024.2007, 38848477 PMC11161857

[70] Sun G Lin K Ai J Zhang W. The efficacy of antivirals, corticosteroids, and monoclonal antibodies as acute Covid-19 treatments in reducing the incidence of long COVID: a systematic review and meta-analysis. Clin Microbiol Infect. (2024) 30:1505–13. doi: 10.1016/J.Cmi.2024.07.006, 39002665

[71] Chow N Tsang C Chan YH Telaga SA Ng L Chung CM . The effect of pre-COVID and post-COVID vaccination on long COVID: a systematic review and meta-analysis. J Infect. (2024) 89:106358. doi: 10.1016/J.Jinf.2024.106358, 39580033

[72] Atieh O Daher J Durieux JC Abboud M Labbato D Baissary J . Vitamins K2 and D3 improve long COVID, fungal translocation, and inflammation: randomized controlled trial. Nutrients. (2025) 17:304. doi: 10.3390/Nu17020304, 39861434 PMC11767688

[73] RECOVERY Collaborative Group. Immunomodulatory therapy in children with paediatric inflammatory multisystem syndrome temporally associated with SARS-CoV-2 (PIMS-TS, MIS-C; recovery): a randomised, controlled, open-label, platform trial. Lancet Child Adolesc Health. (2024) 8:190–200. doi: 10.1016/S2352-4642(23)00316-4 38272046

[74] Gonzalez-Ochoa AJ Szolnoky G Hernandez-Ibarra AG Fareed J. Treatment with sulodexide downregulates biomarkers for endothelial dysfunction in convalescent COVID-19 patients. Clin Appl Thromb Hemost. (2025) 31:10760296241297647. doi: 10.1177/10760296241297647, 39763448 PMC11705351

[75] Shu Y He L Liu C. Impact of anti-fibrotic medications on post-COVID-19 pulmonary fibrosis: a systematic review and meta-analysis. Int J Infect Dis. (2024) 147:107193. doi: 10.1016/J.Ijid.2024.107193, 39094763

[76] Zeraatkar D Ling M Kirsh S Jassal T Shahab M Movahed H . Interventions for the management of long covid (post-covid condition): living systematic review. BMJ. (2024) 387:E081318. doi: 10.1136/Bmj-2024-081318, 39603702 PMC11600537

